# Children’s School-Day Nutrient Intake in Ontario: A Cross-Sectional Observational Study Comparing Students’ Packed Lunches from Two School Schedules

**DOI:** 10.3390/nu14091966

**Published:** 2022-05-08

**Authors:** Lisa J. Neilson, Lesley A. Macaskill, Jonathan M. H. Luk, Navreeti Sharma, Marina I. Salvadori, Jamie A. Seabrook, Paula D. N. Dworatzek

**Affiliations:** 1 School of Food and Nutritional Sciences, Brescia University College at Western University, 1285 Western Road, London, ON N6G 1H2, Canada; lisajneilson@gmail.com (L.J.N.); lmacaski@uwo.ca (L.A.M.); luk.jonathan@gmail.com (J.M.H.L.); navreeti.sharma@gmail.com (N.S.); jseabro2@uwo.ca (J.A.S.); 2 Department of Pediatrics, Faculty of Medicine and Health Sciences, McGill University, 1001 Decarie Boulevard, Montreal, QC H4A 3J1, Canada; marina.salvadori@mcgill.ca; 3 Department of Paediatrics, Schulich School of Medicine and Dentistry, Western University, 1151 Richmond Street, London, ON N6A 3K7, Canada; 4 Department of Epidemiology and Biostatistics, Western University, 1151 Richmond Street, London, ON N6A 3K7, Canada; 5 Human Environments Analysis Laboratory, Faculty of Social Science, Western University, 1151 Richmond Street, London, ON N6A 3K7, Canada; 6 Children’s Health Research Institute, 800 Commissioners Road East, London, ON N6C 2V5, Canada; 7 Lawson Health Research Institute, 750 Base Line Road East, Suite 300, London, ON N6C 2R5, Canada; 8 Schulich Interfaculty Program in Public Health, Schulich School of Medicine & Dentistry, Western University, 1465 Richmond Street, London, ON N6G 2M1, Canada

**Keywords:** elementary school, packed lunches, bag lunches, school lunch, direct food observation, school schedule, nutrient intake, dietary intake

## Abstract

This study compared the caloric and nutrient values of packed lunch contents and consumption in the Balanced School Day (BSD) (two 20 min eating periods) versus the Traditional Schedule (TS) (one 20 min lunch). Foods consumed during school were assessed by direct food observation in 321 grade 3 and 4 students, aged 7–10 years, at 9 BSD and 10 TS elementary schools in Ontario. Packed lunch contents in the BSD were significantly higher than the TS in energy (3128.14 ± 1100.36 vs. 2658.98 ± 951.34 kJ, *p* < 0.001, respectively). Similarly, carbohydrates, total sugar, protein, fat, saturated fatty acids (SFA), calcium, iron, and sodium were significantly higher in the BSD versus TS packed lunches. Correspondingly, students in the BSD consumed significantly more energy, carbohydrates, total sugar, and SFA compared to the TS. Overall, lunches brought by students in the BSD schedule provided more energy across all macronutrients, with only a few micronutrients showing increased amounts, suggesting two 20 min eating opportunities could contribute to excess caloric intake during school, potentially contributing to the prevalence of childhood overweight and obesity in Canada. Furthermore, packed lunches in both schedules had excess amounts of nutrients of concern and much work is needed to ensure that children in Canada receive nutritious lunches at school.

## 1. Introduction

Overweight and obesity continue to be a serious health problem among Canadian children, with approximately one in three children aged 5 to 17 years classified as overweight or obese [1]. Given that a large segment of their day is spent at school, the school environment can be used to positively influence children’s dietary intake, and potentially reduce obesity through health promotion and nutrition policies [2,3].

Many Canadian provinces have school nutrition policies with varying regulations about the type of food and beverages sold to students in elementary and secondary schools [4]. However, these standards do not apply to home-packed lunches. This is particularly important in Canada, as at least 80% of elementary school lunches are packed from home [5] because there is no standardized school meal program [6]. Studies in other jurisdictions that were able to compare home-packed lunches to school-provided meals, found packed lunches to be of low nutritional quality compared to school-provided meals and/or guidelines [7,8,9,10]. Specifically, home-packed lunches have been shown to provide foods containing more energy, sugar, saturated fat, and/or sodium [7,8,11,12,13,14,15,16]. In addition, 70% of energy intake from home-packed lunches has been shown to be from highly processed foods [17]. There are limited data from Canada, but one study found the foods consumed from home-packed lunches, by 10–12-year-old Prince Edward Island (PEI) students, were of poor nutritional value and deemed inadequate when compared to dietary reference intake (DRI) recommendations [18]. In another study, it was concluded that diet quality of foods consumed during school hours required improvement in all age groups [19]. In countries where there is no mandated school meal program, including regulations for delivering healthy food to children at lunch, it is important to identify foods/nutrients that children are bringing and consuming during school hours.

Furthermore, it is important to identify any other factors that may affect the school environment and packed lunch contents and intake. One such factor is a modification to the school schedule. In Ontario, Canada, a modification to the school schedule in many elementary schools was referred to as the Balanced School Day (BSD). In contrast to the traditionally offered breaks (i.e., lunch and two recesses), the BSD provides two 40–50 min breaks, each with 20 min for eating [20,21]. This results in more time dedicated to eating than the 20 min offered during the 60-minute midday break in the traditional elementary school schedule (TS). Many individual schools and entire school boards in Ontario have adopted this new schedule, although the exact number is difficult to quantify as the implementation is not formally mandated nor monitored [20]. Improved learning, dietary intake, and physical activity are often cited as the benefits of the BSD schedule [20,21]; however, limited systematic evaluations supporting or refuting [22] these benefits exist. Considering home-packed lunches have been shown to be high in energy, saturated fat, sodium, and sugar, it is plausible that the additional 20 min eating period provided in the BSD could promote increased low-quality foods/beverages in home-packed lunches. A study comparing students’ lunches in one BSD and one TS school found students in the BSD consumed more beverages; however, they were not able to detect a significant difference in macronutrient or micronutrient intake in students in the two schedules [23]. We found significantly more sugar-sweetened beverages, as well as snack food items, packed in lunches of grades 3 and 4 students in nine BSD vs. ten TS schools [24], and the purpose of this paper is to report on the macronutrient and micronutrient contents and intake of these home-packed lunches in the BSD vs. the TS.

## 2. Materials and Methods

### 2.1. Recruitment and Participants

A representative sample of schools from rural and urban locations with varying levels of socioeconomic risk was obtained from the Thames Valley School Board in Ontario, Canada. Principals and teachers provided support to invite all students in the grade 3 and 4 classes that agreed to participate. Students aged 7–10 years (and their parents) were invited, as they are less likely to have reached the stage of puberty where rapid growth rate could impact food intake and body mass index (BMI) [25]. Participants were excluded if they went home for lunch daily; were on a therapeutic diet; or had a chronic or debilitating condition which may impact their food intake, metabolism, or growth (e.g., Prader-Willi syndrome, diabetes, phenylketonuria).

### 2.2. Direct Observation

Following 10 h of training in food laboratory and classroom environments, research assistants utilized an unobtrusive direct food observation technique (maintaining a 6 ft distance and not interacting with students) to record descriptions and portion sizes of every detectable food and beverage item packed and consumed by participating students on a detailed and directive food observation form, as described previously [24,26]. Research assistants were present at each eating opportunity during one school day to obtain group-level intake. One day is considered adequate when obtaining group-level intake, as opposed to individual-level intake which would require more days of observation [27]. Direct observation, when conducted by trained individuals, has demonstrated accuracy and reliability in a controlled setting such as a school lunchroom or cafeteria [26,27,28,29,30]. To reduce the influence on what was packed in students’ lunches, parents and students were not informed of scheduled data collection dates. Holidays and special school functions were avoided, as typical consumption at school would be disrupted.

### 2.3. Anthropometric Measurements

Height and weight measurements of participating students were taken by a trained observer. A portable stadiometer (Seca Model 213, Seca N. America East, Hanover, MD, USA) and a professional digital scale (Tanita WB-100A, Tanita Arlington Heights, IL, USA) with a portable digital display were utilized. Three measurements of height were taken for each student, as height is more susceptible to error, and the mean value was recorded to the nearest 0.1 cm [31]. To reduce participant discomfort, measurements were taken in a private room, and the results were not disclosed to the participating student, parents, school staff, or other students. BMI was calculated from the two anthropometric measurements, and World Health Organization growth charts were used to determine the corresponding gender specific BMI-for-age Z-score [32].

### 2.4. Parental and School Surveys

A parental survey (completed by the child’s parent) was used to measure additional factors that could contribute to food and beverage items packed and consumed at school to take potential confounding variables into account when assessing the relationship of the BSD to packed lunch contents and intake.

Parental surveys obtained information regarding food neophobia and picky eating. Food neophobia and picky eating have been associated with poor dietary behaviours such as limited fruit and vegetable intake and have the potential to influence packed lunch contents and intake [33,34,35,36,37]. The modified Child Food Neophobia Scale (CFNS) used by Cooke et al. [35] was condensed to promote a high response rate and remain relevant to the school setting [36]. Four of the 6 items on the CFNS were used, with each question using a 5-point scale ranging from ‘strongly disagree’ to ‘strongly agree’; higher scores were associated with higher food neophobia/picky eating. The four items included were: “If my child doesn’t know what is in a food, he or she won’t try it”, “My child is afraid to eat things he or she has never had before”, “My child is very particular about foods he or she will eat”, and “My child will eat almost anything” (reverse scored). This 4-item version showed good internal consistency (Cronbach’s alpha = 0.91) [36].

Two measures of self-reported socioeconomic status, parental educational attainment and household income were also obtained from parental surveys. Parental income and education have been linked to diet quality, with less healthy dietary patterns observed in families with lower educational attainment of parents, while healthy eating and active lifestyles are more common in families with higher incomes and parents with higher educational attainment [38,39]. For parents who declined to provide their income range (24%), a value was imputed based on the median income level of the school their child attended, allowing for the total sample size to be retained.

A school survey was completed by school principals to collect logistic information (e.g., teacher names and contact information, break times, pizza days), as well as 9 items related to the school food environment (e.g., school food retail environment (cafeteria/vending), school food programs (breakfast/snack), packed lunch resources, healthy eating programs). Scores spanned from zero to nine, with higher scores indicative of a healthier school food environment.

A Social Risk Index (SRI) was provided by the School Board and used as a measure of socioeconomic status for each participating school. It incorporated seven risk indicators, using data from the Canadian census, including average household income, lone parents, unemployment, education less than secondary school, moved in last year, non-official languages spoken at home, and newcomers to Canada in the previous five years. Higher scores were indicative of higher risk of disadvantage.

### 2.5. Data and Statistical Analyses

All recorded food and beverage items were entered into ESHA food processor software (Version 10.12.0; ESHA Research, Salem, OR, USA), and two research team members crosschecked all entries with the corresponding hardcopies to ensure accuracy. Food and beverage items were coded with Canadian Nutrient File (CNF) or U.S. Department of Agriculture (USDA) items whenever possible, as manufacturer-provided information often has more incomplete micronutrient data [40,41]. The data were reviewed for extreme values, and appropriate corrections were made when necessary.

Data were analysed using the Statistical Package for Social Sciences (SPSS) (IBM Corp. Released 2012, Version 22.0, Armonk, NY, USA). The significance level was set at *p* < 0.05. Descriptive statistics were produced, by school schedule, for all demographic and socioeconomic characteristics, as well as nutrient values for packed-lunch contents and intake. The chi-squared test for independence was used to compare categorical demographic and socioeconomic data by school schedule. An independent samples *t*-test compared means for normally distributed continuous variables, while a non-parametric Mann–Whitney U test was performed when variables were skewed. Mean values are presented in tables for consistency, with notations to indicate when the Mann–Whitney test was used.

The percentage of CNF and USDA food items with vitamin K and vitamin E values available was less than 60% [40,41]; therefore, these vitamins were excluded from analyses as the lack of data could prevent a complete understanding of students’ intake of these vitamins. The nutrients included in the analyses contained data for more than 70% of food items in the CNF and USDA databases, allowing for a more accurate assessment of intake for these nutrients [40,41].

To determine the adequacy of dietary intake during the school day (approximately a third of daily intake), the proportion of children achieving one-third of available reference standards, by school schedule, was evaluated using the chi-squared test. One-third was chosen as The American Dietetic Association recommends children consume one-third of daily intake in programs lasting 4–7 h [42] and scientific findings have shown that children consume approximately one-third of their daily calories at school [43]. In addition, a one-sample *t*-test (or a one-sample Wilcoxon signed-rank test for skewed data) compared the intake of nutrients, within each schedule, to one-third of an available reference standard; means are presented for consistency. DRIs were used as reference standards, since total nutrient cut-offs for school meal programs do not exist in Canada [6,44]. Mean intakes were compared to the DRI recommendations for 9–13-year-olds, as the mean ± SD age for the participants was 9.12 ± 0.63 years. Estimated Average Requirements (EARs) were used for comparisons when available, because EARs are appropriate for determining the adequacy of group-level intakes [45,46]. When EARs were not available, Adequate Intakes (AIs) were used. The Recommended Daily Allowance (RDA) was used for protein, as it provided a total daily gram value based on a reference body weight. Estimated energy requirements (EERs) for low active 9-year-old male and female children [44,47] were used, because it is estimated that Canadian children are sedentary for 60% of awake hours [48]. Health Canada’s percent daily value for sugar (20%) was used [49].

The energy and nutrient contribution of snack food items was also analysed. A food item was classified as a snack if it was a non-entrée, non-beverage, non-fresh fruit, or vegetable, sweet or savoury item, packaged for consumption in a single sitting [24,50]. For the analysis of nutrients consumed from snack foods, participants (*n* = 25) were omitted from the analysis when no snack foods were consumed.

Multiple regression was used to determine associations between energy packed or consumed, and potential covariates (i.e., BMI z-scores, SRI, neophobia/picky eating, and parental socioeconomic status). Potential co-variates that significantly correlated with the dependent variable (energy packed or consumed) were included in the regression models. Bivariate correlations were also conducted for all potential co-variates to ensure cases of multicollinearity were not simultaneously included in the models.

## 3. Results

A total of 321 grade 3 and 4 students, aged 7–10 years, were observed in 19 elementary schools in the Thames Valley District School Board. Of the 19 participating schools, 9 were following the Balanced School Day (*n* = 153), whereas 10 were adhering to the Traditional Schedule (*n* = 168). The final sample size represented a 44% response rate (after omitting data for 18 students who were unavailable for observations), as 731 children were invited to participate.

There were no statistically significant differences between children in the BSD compared to those in the TS with respect to sex, grade, school location (rural vs. urban), neophobia, or BMI z-score (Table 1). Children in the BSD schedule were slightly older (9.25 vs. 9.00 years, *p* < 0.001, respectively) compared to children in the TS. More TS children had parents with post-secondary education (77.5 vs. 62.8%; *p* = 0.01). Similarly, TS children had higher family incomes (CAD 83,296 ± 36,766)) than children in the BSD schedule (CAD 60,424 ± 30,959) (*p* < 0.001)). The mean SRI for BSD school communities was significantly higher than TS schools, indicating a higher risk of disadvantage in BSD schools. Conversely, a significantly higher school food environment score occurred in BSD schedule schools compared to the TS, suggesting the school food environments in BSD schools were more likely to foster healthy eating behaviours.

Students in the BSD schedule had significantly greater energy, carbohydrate, sugar, percent energy from sugar, protein, fat, saturated fatty acids, sodium, calcium and iron from foods packed in their lunches than TS students (Table 2). Children did not consume all foods packed in their lunches; nevertheless, consumption of energy, carbohydrates, sugar, percent energy from sugar, and saturated fatty acids remained significantly higher for BSD schedule students than TS (Table 3). The percentage of energy intake from protein was significantly lower for BSD students compared to TS schedule students (10.37% vs. 11.61%, *p* = 0.02, respectively). 

Table 4 illustrates the proportion of children whose intake achieved one-third of population recommendations, by school schedule. There were no significant differences between school schedules in the proportion of children meeting recommendations (*p* > 0.05). Notably, less than 7% of students in both schedules met one-third of the DRI recommendations for fibre, potassium, and vitamin D and less than 40% of students met the recommendations for calcium, phosphorus, magnesium, zinc, vitamin A, and folate.

The mean intake of nutrients consumed by students in each schedule was also compared to one-third of the DRI or population recommendation (Table 5). In both schedules, the intakes of calcium, phosphorus, zinc, vitamin A (females), and vitamin D fell below recommendations (i.e., one-third of EAR). Fibre and potassium intakes in both schedules were also below one-third of the recommended AI, while sodium exceeded one-third of daily recommendations. At the same time, intake of carbohydrates, iron, thiamin, riboflavin, niacin, vitamin B6, vitamin B12, and vitamin C exceeded one-third of EAR recommendations in both schedules. Protein intake was also above one-third RDA recommendations in both schedules. Total sugar intake surpassed one-third of the % daily value. Only folate and female energy intake in both schedules were not significantly different from nutrient recommendations. In comparing the two schedules, the amount of magnesium consumed in the TS and the intake of energy and vitamin A by male TS students were significantly below recommendations, while intake of these nutrients by BSD students adequately met recommendations.

Nutrient intakes from snack food consumption are shown in Table 6 by school schedule. Snacks consumed in the BSD schedule provided significantly more energy, carbohydrates, sugar, fat, saturated fat, and sodium than in the TS. Snacks also provided significantly higher intakes of protein, calcium, iron, potassium, zinc, vitamin A, vitamin B6 and vitamin D when compared to the TS.

Potential co-variates that were significantly associated with energy packed (school schedule, BMI Z-score, and school food environment score) or consumed (school schedule, BMI Z-score, SRI, and school location) were included in multiple regression models. Parental income, parental educational attainment, neophobia/picky eating, age, grade, and sex were not significantly correlated with energy packed or eaten and were not included in the models (data not shown). In addition to school schedule predicting energy in packed lunches (B = 100.12, β = 0.20, Adjusted R^2^ = 0.10, *p* = 0.01), BMI Z-score was also predictive (B = 44.66, β = 0.24, *p* = 0.01), while the school food environment score was not. Attending the BSD and higher BMI Z-scores were predictive of more energy being packed in students’ lunches. For energy consumed, school schedule (B = 50.00, β = 0.12, Adjusted R^2^ = 0.12, *p* =0.04), BMI Z-score (B = 43.52, β = 0.29, *p* < 0.001), and school SRI were predictive (B = −74.02, β = −0.12, *p* = 0.037), while school location was not. Attending a school on the BSD schedule and higher BMI Z-scores were predictive of higher energy consumption, whereas a higher SRI, corresponding to school populations at a higher risk for disadvantage, was predictive of lower energy intake. In both of these models, the Adjusted R^2^ indicates that the models only account for 10 and 12% of the variation in calories packed and consumed, respectively. Nevertheless, attending the BSD schedule remained predictive of higher energy consumed from packed lunches, when co-variates were included in the model.

## 4. Discussion

Home-packed lunches brought to school by children in the BSD schedule contained more energy, carbohydrate, sugar, protein, fat, saturated fatty acids, sodium, calcium, iron, and percent energy from sugar, and resulted in higher intakes of energy, carbohydrates, saturated fatty acids, sugar and percent energy from sugar compared to those in the TS. The total amount of protein consumed was similar between schedules; however, due to higher consumption of other macronutrients in the BSD, the percentage of energy from protein was lower in the BSD schedule.

The findings of this study do not correspond to the only other study of student packed-lunch intake in the BSD [23], in which no difference was found in the macronutrient and micronutrient composition of the foods and beverages consumed compared to the TS. The reasons for these differences could include a larger sample size of both students and schools, and the rigorous direct food observation methodology and nutrient quantification used to assess dietary intake in the present study.

The overall quality of home-packed lunches in both schedules was poor when compared to recommended intakes. Home-packed lunch intake failed to meet fibre, potassium, vitamin D, calcium, zinc, and phosphorus recommendations. Consumption of vitamin A by females in both schedules was also below recommendations. At the same time, consumption of carbohydrates, sugars, and sodium exceeded recommendations in both school schedules. This is concerning as Health Canada recommends limiting intake of sugar and sodium, as they are not conducive to a healthy population when consumed in excess [51]. Consumption of thiamin, riboflavin, niacin, iron, protein, and vitamins B6, B12, and C also surpassed recommendations. Fortification of white flour with thiamin, riboflavin, niacin, iron and folic acid has been mandatory in Canada since 1998 or earlier, to support adequate intake of these vitamins from the food supply [52]. Fortification practices in Canada likely contributed to adequate intakes of these nutrients; however, considering the low fibre intakes, refined low-fibre carbohydrates are likely food sources. Taylor et al. [18] found home-packed lunches of grade 5 and 6 PEI students to be lacking in magnesium, potassium, zinc, vitamin A, D, C, B6, folate and fibre when compared to DRIs. This is similar to the findings of the present study, with the exception of magnesium, folate, vitamin B6 and vitamin C. Adequate magnesium intake in the BSD schedule may be due to more servings of milk and alternatives consumed from BSD home-packed lunches [24]. Considering the poor vitamin D and calcium intake in the BSD schedule, it would be reasonable to assume cheese strings and yogurt tubes contributed to the higher milk and alternative servings and magnesium intakes in the BSD. Cheese is often considered a healthy snack option for kids; however, cheese may be providing greater amounts of sodium and saturated fat, and less vitamin D than an equal Canada’s Food Guide serving of milk. Furthermore, flavoured yogurt tubes also contribute a considerable amount of energy from sugar relative to their small size. Consumption of vitamin C in this study was likely above recommendations due to the popularity of sugar-sweetened fruit drinks/cocktails, as well as gummy-type snacks, that may include manufacturer-added vitamin C [24]. In fact, snack items were common in home-packed lunches from both schedules when compared to other food categories and may also have contributed to higher intakes of carbohydrate, sugar, fat, and sodium. Moreover, intake from sugar-sweetened beverages was greater than that from 100% fruit juice in both schedules, whereas vegetable and fruit intake was poor [24], all contributing to high sugar intakes. 

The type of dietary pattern observed in the present study does not reflect public health messages focused on limiting sugar, fat, saturated fat, and sodium intakes [51,53]. Rising intakes of sugar, fat, saturated fat, and sodium have been recognized as factors contributing to overweight in childhood, which in turn increases a child’s risk for morbidities and premature mortality later in life [54,55,56]. Overall intake, as well as increased consumption of snack items, contributed to higher amounts of these nutrients in the BSD schedule when compared to the TS, even though snack items also contributed some protein and micronutrient benefits. Snack frequency has been positively associated with an increase in both healthy and less healthy foods that contribute to daily intake of macronutrients and some micronutrients in both adults and children [57,58]. However, it is difficult to compare literature surrounding snack intake and snack frequency, as the definition of a snack is not consistent between research studies. Despite the possibility of a few snack foods containing valuable nutrient content, the consumption of a greater number of snack items in the BSD schedule contributed additional carbohydrates, sugar, fat, saturated fat, sodium and energy, and this could be a concern for children who are on the BSD schedule.

Children in the BSD schedule consumed more energy from their home-packed lunch than children in the TS. The energy intake of both genders in the BSD schedule did not significantly differ from recommendations, but intakes were above recommended amounts. A sustained additional daily consumption of 220 kJ/53 kcal per day, as we observed in the BSD vs. TS, has been associated with weight gain [59]. Thus, it is plausible that increased energy consumption, without an increase in expenditure, in the BSD schedule has the potential to contribute to the already elevated childhood overweight and obesity rates in Canada. One study, comparing the number of steps taken by students during breaks from instructional time in the BSD schedule compared to the TS, found students in the BSD schedule took fewer steps [22], despite claims that the BSD can result in increased participation in physical activity [20].

After controlling for potential covariates, school schedule continued to be a predictor of energy packed and consumed while at school. The BSD schedule was predictive of having more energy packed in home-packed lunches, and more energy consumed while at school. BMI Z-score was also predictive of energy packed and consumed while at school. As this study was cross-sectional, it is not possible to determine which came first—the higher energy in packed lunches or the elevated BMIs. While a low income has been associated with a nutrient-poor diet, low in fibre, potassium, and vitamins A and C, and high in fat and saturated fat [60,61], in this study parental income was not related to energy packed or eaten. Rather, SRI, which included additional risk factors for disadvantage, was predictive of energy consumed.

Consumption of total sugar was high in the present study (38 g TS and 47 g BSD), with intakes in both schedules exceeding one-third (33.3 g) of the percent daily value [49]. Unfortunately, the sugar values present on nutrition facts tables in Canada provide data on total sugars only [62]. Similarly, the food composition database used in the present study does not differentiate between added and total sugars [40]. Nevertheless, it is quite likely that added sugars (refined sugars added to a food item during cooking or manufacturing [63]) were consumed based on the nutrient profile of snacks consumed and previous data estimating the percent intake of added sugars in Canadian diets [64]. In children, added sugars have been found to be positively associated with elevated diastolic blood pressure and triglycerides [65].

A few limitations exist in the present cross-sectional study. Causation cannot be assumed with a cross-sectional study; however, potential confounders were taken into consideration using regression analyses. The response rate of 44% may be a limitation indicative of self-selection; however, it has been shown that that there is little evidence of bias from low response rates when using multivariate statistics [66], which we did. Additionally, response rates are often hindered by the requirement of the school board to send packages home and for parents to find them and respond in a timely fashion. This is a challenge in all school recruitment studies whereby we are required to comply with ethics boards. Fat consumption, and therefore energy intake, may have been underestimated, as research assistants only recorded fillings and spreads, such as butter/mayonnaise on a sandwich, if they were visible. Similarly, only visible food was recorded; thus, food items not removed from the lunch bag during any food consumption times were not able to be recorded as packed. The reader should take into account that we compared the group-level intake (mean ± SD age, 9.12 ± 0.63 years) to the 9–13-year-old DRIs when some of the children were slightly below this age category. Finally, school day observations only capture about a third of daily food consumption, and total daily food intake may be improved with healthier choices at other times in the day [67,68]. It is important to note, however, that after-school food choices by this age group have been found to provide additional energy with few nutrients [69].

## 5. Conclusions

Lunches brought by students in the BSD schedule provided more energy across all macronutrients, with only a few micronutrients showing increased amounts. These findings suggest that a seemingly beneficial change in the school schedule, resulting in two 20 min eating opportunities, could result in unintended negative outcomes by contributing to excess energy intake during school. This could ultimately lead to weight gain and contribute to the already high childhood overweight and obesity rates in Canada. More research is needed to determine the long-term impact of the BSD schedule on dietary and anthropometric measures. More importantly, interventions are warranted in both schedules to promote the packing and consumption of nutrient dense foods in students’ lunches to decrease the intake of high-fat, high-sodium, and high-sugar foods. Ultimately, population-level interventions that provide appealing nutrient-dense lunches at school, i.e., universal school food programs, would provide opportunities to improve the nutrient intake of all school children in Canada [6].

## Figures and Tables

**Table 1:** Participant characteristics by school schedule.

**Characteristics**	**Total**		**BSD**		**TS**		***p*** **Value ^1^**
	***n***	**%**	***n***	**%**	***n***	**%**	
Number of participants	321	100	153	48	168	52	
Sex							0.95
Male	160	49.8	76	49.7	84	50.0	
Female	161	50.2	77	50.3	84	50.0	
Grade							0.18
Three	172	53.6	76	49.7	96	57.1	
Four	149	46.4	77	50.3	72	42.9	
Highest education attained by parent ^2^							0.01
Less than post-secondary	90	29.5	54	37.4	36	22.5	
Post-secondary	215	70.5	91	62.8	124	77.5	
School Location							0.41
Rural	133	41.4	67	43.8	66	39.3	
Urban	188	58.6	86	56.2	102	60.7	
**Characteristics**	**Mean**	**SD**	**Mean**	**SD**	**Mean**	**SD**	***p*** **Value ^1^**
Age (years)	9.12	0.63	9.25	0.59	9.00	0.63	<0.001
Parental income (CAD)	72,394	35,939	60,424	30,959	83,296	36,766	<0.001
School food environment score ^3^	5.7	1.2	6.6	0.9	5.0	0.8	<0.001
Food neophobia score ^4^	11.4	4.3	11.3	4.4	11.5	4.3	0.78
BMI Z-Score	0.6	1.4	0.8	1.5	0.5	1.3	0.09
Social Risk Index ^5^	0.2	0.4	0.3	0.3	0.1	0.4	<0.001

BSD, balanced school day; TS, traditional schedule. ^1^ Differences assessed using χ^2^ test for categorical variables and an independent samples *t*-test for continuous variables. ^2^ Total sample size is 305 due to ‘decline to answer’ responses (TS = 160, BSD = 145). ^3^ Scores ranged from 0 to 9, with higher scores indicative of a healthier school food environment. ^4^ Scores ranged from 4 to 20, with higher scores indicative of neophobia. ^5^ Scores ranged from −0.67 to 1.2, with higher scores indicative of greater risk.

**Table 2:** Nutrients packed by school schedule.

Nutrient	Total (*n* = 320 ^1^)		BSD (*n* = 151 ^1^)		TS (*n* = 168)		*p* Value ^2^
	Mean	SD	Mean	SD	Mean	SD	
Energy (kJ)	2885.90	1046.88	3128.14	1100.36	2658.98	951.34	<0.001
Energy (kcal)	688.96	250.96	747.64	262.99	635.51	227.38	<0.001
Carbohydrates (g)	107.29	39.95	116.62	39.99	98.79	38.08	<0.001
% Energy from Carbohydrates	63.25	12.42	63.81	11.87	62.74	12.92	0.44
Total Sugar (g)	47.56	26.10	55.16	27.03	40.64	23.23	<0.001
% Energy from Total Sugar	27.97	13.60	30.50	13.77	25.67	13.05	0.001
Fibre (g)	5.76	2.92	6.03	2.93	5.52	2.90	0.12
Protein (g)	18.48	9.09	19.55	9.65	17.51	8.45	0.046
% Energy from Protein	10.89	4.54	10.44	4.20	11.30	4.80	0.09
Fat (g)	22.03	12.61	24.10	13.48	20.14	11.49	0.01
% Energy from Fat	27.71	10.54	27.73	10.00	27.68	11.03	0.97
SFA (g)	7.68	5.03	8.57	5.55	6.87	4.36	<0.01
% Energy from SFA	9.64	5.01	9.80	4.86	9.50	5.16	0.59
Sodium (mg)	1014.06	506.79	1113.48	550.89	923.53	445.65	0.001
Calcium (mg)	263.87	181.61	289.71	205.25	240.34	153.91	0.02
Iron (mg)	4.54	2.31	4.94	2.30	4.17	2.27	<0.01
Phosphorus (mg)	336.48	179.59	350.69	198.02	323.54	160.50	0.18
Magnesium (mg)	72.93	38.27	75.97	38.87	70.17	37.62	0.18
Potassium (mg)	653.08	332.37	685.01	335.93	623.99	327.38	0.10
Zinc (mg)	2.35	1.53	2.45	1.36	2.27	1.67	0.31
Vitamin A RAE (µg)	135.95	197.35	158.45	226.34	115.45	164.66	0.09 *^3^*
Thiamin (mg)	0.52	0.32	0.56	0.36	0.49	0.28	0.06
Vitamin B12 (µg)	0.73	0.72	0.77	0.71	0.70	0.73	0.39 ^3^
Folate DFE (µg)	97.87	75.11	104.99	71.61	91.39	77.80	0.10
Riboflavin (mg)	0.54	0.35	0.58	0.39	0.50	0.30	0.06
Niacin NE (mg)	8.65	5.12	9.17	5.60	8.17	4.58	0.08
Vitamin B6 (mg)	0.42	0.35	0.44	0.39	0.40	0.30	0.26
Vitamin C (mg)	45.78	50.34	45.85	46.23	45.73	53.95	0.47 ^3^
Vitamin D (µg)	0.41	0.91	0.44	0.78	0.37	1.02	0.19 ^3^

BSD, balanced school day; TS, traditional schedule; SFA, saturated fatty acids; RAE, retinol activity equivalents; DFE, dietary folate equivalents; NE, niacin equivalents. ^1^ Total sample size was reduced to 320 because one student in the BSD had no packed lunch (*n* = 152), but did consume emergency food received from the school. ^2^ Differences assessed using independent samples *t*-test, except where noted. ^3^ Differences assessed using Mann–Whitney U-test for skewed data; for consistency, data are presented as means.

**Table 3:** Nutrients consumed by school schedule.

Nutrient	Total (*n* = 321)		BSD (*n* = 153)		TS (*n* = 168)		*p* Value ^1^
	Mean	SD	Mean	SD	Mean	SD	
Energy (kJ)	2421.50	914.41	2541.92	987.20	2318.14	827.29	0.03
Energy (kcal)	579.54	218.09	607.53	235.95	554.05	197.73	0.03
Carbohydrates (g)	90.46	35.10	95.30	37.05	86.06	32.73	0.02
% Energy from Carbohydrates	63.62	13.01	64.39	13.16	62.92	12.87	0.31
Total Sugar (g)	42.08	22.55	46.91	24.41	37.68	19.78	<0.001
% Energy from Total Sugar	30.07	14.80	32.45	15.77	27.91	13.54	0.01
Fibre (g)	4.83	2.59	4.84	2.62	4.82	2.56	0.93
Protein (g)	15.89	8.22	15.87	8.16	15.91	8.31	0.96
% Energy from Protein	11.02	4.81	10.37	4.41	11.61	5.09	0.02
Fat (g)	18.29	10.51	19.40	11.45	17.27	9.49	0.07
% Energy from Fat	27.24	10.62	27.31	10.79	27.17	10.50	0.91
SFA (g)	6.55	4.35	7.14	4.92	6.00	3.39	0.02
% Energy from SFA	9.70	5.09	9.91	5.29	9.51	4.92	0.49
Sodium (mg)	824.29	461.47	865.36	500.87	786.89	420.43	0.13
Calcium (mg)	239.81	164.90	245.14	174.97	234.96	155.54	0.58
Iron (mg)	3.77	2.19	4.01	2.26	3.55	2.11	0.06
Phosphorus (mg)	301.10	164.70	297.84	170.91	304.06	159.29	0.74
Magnesium (mg)	62.93	32.19	64.16	32.98	61.82	31.50	0.52
Potassium (mg)	579.45	313.85	581.54	330.90	577.53	298.45	0.91
Zinc (mg)	2.06	1.45	2.07	1.26	2.06	1.61	0.94
Vitamin A RAE (µg)	122.87	158.04	135.68	175.89	111.20	139.34	0.63 ^2^
Thiamin (mg)	0.43	0.28	0.44	0.29	0.42	0.27	0.56
Vitamin B12 (µg)	0.69	0.65	0.66	0.59	0.71	0.70	0.51
Folate DFE (µg)	81.15	69.57	84.24	63.92	78.33	74.42	0.45
Riboflavin (mg)	0.47	0.28	0.46	0.27	0.48	0.29	0.57
Niacin NE (mg)	7.18	4.28	7.17	4.18	7.20	4.39	0.95
Vitamin B6 (mg)	0.35	0.27	0.35	0.27	0.35	0.28	0.89
Vitamin C (mg)	38.59	43.43	39.71	43.68	37.57	43.31	0.62 ^2^
Vitamin D (µg)	0.57	1.07	0.44	0.78	0.68	1.27	0.35 ^2^

BSD, balanced school day; TS, traditional schedule; SFA, saturated fatty acids; RAE, retinol activity equivalents; DFE, dietary folate equivalents; NE, niacin equivalents. ^1^ Differences assessed using an independent *t*-test, except where noted. ^2^ Differences assessed using Mann–Whitney U-test for skewed data; for consistency, data are presented as means.

**Table 4:** Proportion of children consuming one-third of recommendations, by school schedule.

Nutrient	One-Third of Recommended Nutrient Intake ^1^	BSD (*n* = 153)		TS (*n* = 168)		*p* Value ^2^
		No.	% Meeting Recommendations	No.	% Meeting Recommendations	
Energy (kJ)	7475.8, M; 6945.4, F	82	53.6	73	43.5	0.07
Energy (kcal)	595.7, M; 553.33, F	82	53.6	73	43.5	0.07
Carbohydrates (g)	33.3	148	96.7	163	97.0	0.88
Total Sugar (g)	33.3	105	68.6	100	59.5	0.09
Fibre (g)	10.3, M; 8.7, F	8	5.2	10	6.0	0.78
Protein (g)	11.3	103	67.3	114	67.9	0.92
Sodium (mg)	500.0	120	78.4	122	72.6	0.23
Calcium (mg)	366.7	31	20.3	31	18.5	0.68
Iron (mg)	2.0	125	81.7	134	79.8	0.66
Phosphorus (mg)	351.7	50	32.7	67	39.9	0.18
Magnesium (mg)	66.7	61	39.9	65	38.7	0.83
Potassium (mg)	1500.0	2	1.3	0	0.0	0.14
Zinc (mg)	2.3	57	37.3	54	32.1	0.34
Vitamin A RAE (µg)	148.3, M; 140.0, F	47	30.7	48	28.6	0.67
Thiamin (mg)	0.2	119	77.8	126	75.0	0.56
Vitamin B12 (µg)	0.5	79	51.6	97	57.7	0.27
Folate DFE (µg)	83.3	58	37.9	62	36.9	0.85
Riboflavin (mg)	0.3	120	78.4	127	75.6	0.55
Niacin NE (mg)	3.0	133	86.9	147	87.5	0.88
Vitamin B6 (mg)	0.3	82	53.6	96	57.1	0.52
Vitamin C (mg)	13.0	93	60.8	98	58.3	0.66
Vitamin D (µg)	3.3	0	0.0	4	2.4	0.06

BSD, balanced school day; TS, traditional schedule; no., number of students meeting recommendations; M, male; F, female; RAE, retinol activity equivalents; DFE, dietary folate equivalents; NE, niacin equivalents. ^1^ One-third of the Estimated Average Requirement (EAR) where available for 9–13-year-old children. Energy intake was compared to gender specific Estimated Energy Requirement (EER) values calculated for the sample. Total sugar was compared to Health Canada’s % Daily Value. Protein intakes were compared to Recommended Daily Intake (RDA) values. Fibre, sodium, and potassium intakes were compared to the appropriate Adequate Intake (AI) value. ^2^ Differences assessed using χ^2^ test.

**Table 5:** Comparison of mean intake in each school schedule to one-third of recommendations.

Nutrient	One-Third of Recommended Nutrient Intake ^1^	BSD (*n* = 153)				TS (*n* = 168)			
		No.	Mean	SD	*p* Value ^2^	No.	Mean	SD	*p* Value ^2^
Energy (kcal/d)	595.7, M	77	644.93	220.49	0.054	84	546.61	186.79	0.02
	553.3, F	76	569.65	246.32	0.565	84	561.48	208.95	0.72
Carbohydrates (g/d)	33.3	153	95.30	37.05	<0.001	168	86.06	32.73	<0.001
Total Sugar (g/d)	33.3	153	46.91	24.41	<0.001	168	37.68	19.78	0.01
Fibre (g/d)	10.3, M	77	5.19	2.69	<0.001	84	4.44	2.58	<0.001
	8.7, F	76	4.50	2.51	<0.001	84	5.20	2.51	<0.001
Protein (g/d)	11.3	153	15.87	8.16	<0.001	168	15.91	8.31	<0.001
Sodium (mg/d)	500.0	153	865.36	500.87	<0.001	168	786.89	420.43	<0.001
Calcium (mg/d)	366.7	153	245.14	174.97	<0.001	168	234.96	155.54	<0.001
Iron (mg/d)	2.0	153	4.01	2.26	<0.001	168	3.55	2.11	<0.001
Phosphorus (mg/d)	351.7	153	297.84	170.91	<0.001	168	304.06	159.29	<0.001
Magnesium (mg/d)	66.7	153	64.16	32.98	0.347	168	61.82	31.50	0.048
Potassium (mg/d)	1500.0	153	581.54	330.90	<0.001	168	577.53	298.45	<0.001
Zinc (mg/d)	2.3	153	2.07	1.26	0.012	168	2.06	1.61	0.03
Vitamin A RAE (µg/d)	148.3, M	77	143.82	174.12	0.061 ^3^	84	95.43	92.72	<0.001 ^3^
	140.0, F	76	127.45	178.43	0.007 ^3^	84	126.97	170.34	0.001 ^3^
Thiamin (mg/d)	0.2	153	0.44	0.29	<0.001	168	0.42	0.28	<0.001
Vitamin B12 (µg/d)	0.5	153	0.66	0.59	0.001	168	0.71	0.70	<0.001
Folate DFE (µg/d)	83.3	153	84.24	63.92	0.861	168	78.33	74.42	0.39
Riboflavin (mg/d)	0.3	153	0.46	0.27	<0.001	168	0.48	0.29	<0.001
Niacin NE (mg/d)	3.0	153	7.17	4.18	<0.001	168	7.20	4.39	<0.001
Vitamin B6 (mg/d)	0.3	153	0.35	0.27	<0.001	168	0.35	0.28	<0.001
Vitamin C (mg/d)	13.0	153	39.71	43.68	<0.001 ^3^	168	37.57	43.31	<0.001 ^3^
Vitamin D (µg/d)	3.3	153	0.44	0.78	<0.001 ^3^	168	0.68	1.27	<0.001 ^3^

BSD, balanced school day; TS, traditional schedule; no., number of students meeting recommendations; M, male; F, female; RAE, retinol activity equivalents; DFE, dietary folate equivalents; NE, niacin equivalents. ^1^ One-third of the Estimated Average Requirement (EAR) where available for 9–13-year-old children. Energy intake was compared to gender specific Estimated Energy Requirement (EER) values calculated for the sample. Total sugar was compared to Health Canada’s % Daily Value. Protein intakes were compared to Recommended Daily Intake (RDA) values. Fibre, sodium, and potassium intakes were compared to the appropriate Adequate Intake (AI) value. ^2^ Differences assessed using a one-sample *t*-test, except where noted. ^3^ Differences assessed using a one-sample Wilcoxon signed rank test; for consistency data are presented as means.

**Table 6:** Nutrients consumed from snack items by school schedule.

Nutrient	Total (*n* = 296 ^1^)		BSD (*n* = 148 ^2^)		TS (*n* = 148 ^3^)		*p* Value ^4^
	Mean	SD	Mean	SD	Mean	SD	
Energy (kJ)	1019.14	705.51	1127.65	732.20	920.33	667.23	0.01
Energy (kcal)	243.58	168.62	269.52	175.00	219.96	159.47	0.01
Carbohydrates (g)	37.87	27.03	41.30	28.19	34.75	25.61	0.03
% Energy from Carbohydrates	63.78	16.42	62.91	17.78	64.65	15.07	0.36
Total Sugar (g)	14.48	13.83	17.31	15.14	11.91	11.99	<0.001 ^5^
% Energy from Total Sugar	25.16	19.25	27.25	18.71	23.07	19.61	0.06
Fibre (g)	1.53	1.47	1.60	1.55	1.47	1.41	0.43
Protein (g)	4.20	3.53	4.73	3.80	3.71	3.20	0.01
% Energy from Protein	6.92	4.16	7.12	4.87	6.71	3.33	0.40
Fat (g)	8.73	7.35	9.89	7.83	7.67	6.74	0.01
% Energy from Fat	30.69	14.48	31.52	15.43	29.87	13.64	0.33
SFA (g)	3.02	2.93	3.43	3.14	2.65	2.68	0.02 ^5^
% Energy from SFA	10.76	7.81	10.89	8.05	10.63	7.64	0.78
Sodium (mg)	241.55	204.09	272.89	212.15	213.00	192.67	0.01
Calcium (mg)	78.28	87.69	91.83	93.92	65.95	79.91	<0.01 ^5^
Iron (mg)	1.45	1.49	1.67	1.74	1.25	1.20	0.02 ^5^
Phosphorus (mg)	102.08	91.36	112.05	95.09	93.00	87.12	0.06
Magnesium (mg)	22.39	19.77	24.64	20.72	20.33	18.70	0.05
Potassium (mg)	149.16	150.96	166.94	153.48	132.97	147.22	0.01 ^5^
Zinc (mg)	0.61	0.55	0.70	0.59	0.52	0.52	<0.01 ^5^
Vitamin A RAE (µg)	23.04	43.04	23.77	40.06	22.39	45.70	0.04 ^5^
Thiamin (mg)	0.13	0.17	0.15	0.19	0.12	0.14	0.07 ^5^
Vitamin B12 (µg)	0.16	0.22	0.18	0.22	0.14	0.21	0.09 ^5^
Folate DFE (µg)	19.69	23.71	19.37	22.45	19.99	24.87	0.31 ^5^
Riboflavin (mg)	0.15	0.14	0.17	0.14	0.14	0.14	0.05 ^5^
Niacin NE (mg)	1.89	1.76	2.05	1.80	1.74	1.71	0.12
Vitamin B6 (mg)	1.10	0.12	0.11	0.14	0.08	0.11	0.01 ^5^
Vitamin C (mg)	3.55	8.56	3.21	7.16	3.85	9.67	0.17 ^5^
Vitamin D (µg)	0.03	0.10	0.41	0.13	0.17	0.06	0.047

BSD, balanced school day; TS, traditional schedule; SFA, saturated fatty acids; RAE, retinol activity equivalents; DFE, dietary folate equivalents; NE, niacin equivalents. ^1^ Total sample size reduced to 296, from 321, as some students did not consume a snack. ^2^ Total sample size reduced to 148, from 153, as some students did not consume a snack. ^3^ Total sample size reduced to 148, from 168, as some students did not consume a snack ^4^ Differences assessed using independent samples *t*-test, except where noted. ^5^ Differences assessed using Mann–Whitney U-test for skewed data; for consistency, data are presented as means.

## Data Availability

The data presented in this study are available on request from the corresponding author.
